# Benign Mesothelial Proliferations of the Tunica Vaginalis Testis

**DOI:** 10.3390/clinpract13050101

**Published:** 2023-09-15

**Authors:** Claudia Manini, Estíbaliz López-Fernández, Nicola Cruciano, Alessandro Comandone, José I. López

**Affiliations:** 1 Department of Pathology, San Giovanni Bosco Hospital, ASL Città di Torino, 10154 Turin, Italy; claudia.manini@aslcittaditorino.it; 2 Department of Sciences of Public Health and Pediatrics, University of Turin, 10124 Turin, Italy; 3 FISABIO Foundation, 46020 Valencia, Spain; estibaliz.lopez@universidadeuropea.es; 4 Faculty of Health Sciences, European University of Valencia, 46023 Valencia, Spain; 5 Department of Urology, Maria Vittoria Hospital, ASL Città di Torino, 10144 Turin, Italy; nicola.cruciano@aslcittaditorino.it; 6 Department of Medical Oncology, San Giovanni Bosco Hospital, ASL Città di Torino, 10154 Turin, Italy; alessandro.comandone@aslcittaditorino.it; 7 Biomarkers in Cancer Unit, Biocruces-Bizkaia Health Research Institute, 48903 Barakaldo, Spain

**Keywords:** well-differentiated papillary mesothelioma, florid mesothelial hyperplasia, pseudotumor, differential diagnosis

## Abstract

The correct diagnosis of mesothelial proliferations is a classic problem for pathologists, and one which has important clinical implications. A significant number of such cases appear associated with recurrent hydrocele, as an irritative/reactive response to this condition. The morphological spectrum of mesothelial lesions in this topography is broad, and a set of benign conditions may appear, sometimes with florid gross features and cytologic pseudo-atypia. Here, we present two different examples in which malignancy was initially considered in the differential diagnosis.

## 1. Introduction

Diagnosing some mesothelial lesions may be a problem even for experienced pathologists. In fact, this issue receives considerable attention in textbooks and scientific journals [1,2]. The problem can be summarized in two classic dilemmas: Is this a benign lesion or a malignant tumor? And if it is overtly malignant, is it a mesothelioma or not? The latter doubt is particularly frequent in the pleura, where adenocarcinomas and other epithelioid-appearing tumors of diverse origin can often occur. Many of these diagnostic problems can be accentuated if the sample biopsy is small, the lesion is not well represented, or a stromal reaction in the form of desmoplasia and/or inflammation darkens the general picture. To make matters worse, technical problems, difficult-to-access locations, inexperience, and artifacts provoked by the surgical procedure add further complications. As a result, the diagnosis may not be conclusive, and patients must go for a second biopsy. 

The differential diagnosis of mesothelial proliferations has been extensively reviewed [1,3,4,5]. In particular, immunohistochemistry [4,6,7,8,9,10,11] provides reliable information for distinguishing between benign and malignant proliferations. Molecular studies [4,12] also help in their differential diagnosis. These characteristics, coupled with a critical look at the clinical history, help in making a correct diagnosis. The issue has also medical–legal implications due to the well-known relationship between malignant mesothelioma and asbestos exposure.

Our aim here is to revisit the morphological and immunohistochemical characteristics of benign mesothelial proliferations by taking two typical examples as a model of pseudo-malignant lesions appearing in the tunica vaginalis testis associated with hydrocele.

## 2. Case Studies

### 2.1. Case 1

**Figure 1:**
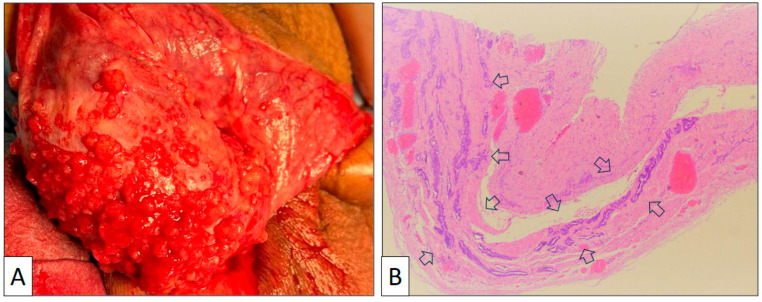
(**A**) Macroscopic view of the tunica vaginalis showing multiple small exophytic nodules on the surface. (**B**) Panoramic view of the tunica vaginalis showing glandular structures with major axis parallel to the wall and no stromal reaction or wall thickening (arrows) (original magnification, ×40).

An 80-year-old male with obesity, type 2 diabetes, and umbilical and inguinal hernia consulted his urologist due to scrotal enlargement. The patient referred to similar previous episodes with spontaneous regression. A physical examination revealed a giant hydrocele with bilateral involvement needing urgent intervention. A blood analysis was unremarkable. On surgery, the tunica vaginalis showed a rough congestive surface (Figure 1A).

**Figure 2:**
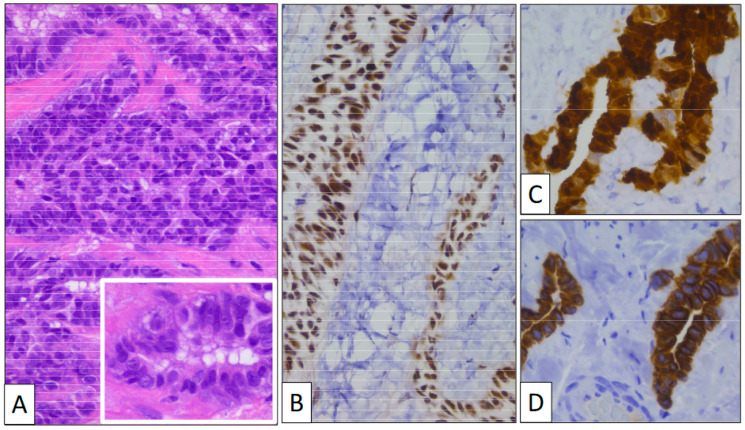
(**A**) Glandular structures growing into the tunica vaginalis (original magnification, ×100). Proliferating cells display a monotonous cuboidal morphology (inset, original magnification, ×400). An immunohistochemical study shows a positive reaction with BAP-1 (**B**), CK7 (**C**), and Calretinin (**D**) (original magnification, ×400 and ×250).

The surgical specimen consisted of an irregular fragment of tunica vaginalis measuring 3 × 4 × 0.5 cm. The thickness of the piece was not significantly enlarged, and no tumor growth was detected with the naked eye. Under the microscope, the low-power view showed a proliferation of tubular structures oriented parallel to the surface with no significant associated stromal reaction or inflammation (Figure 1B). Vascular congestion was also seen. Under a high-power view (Figure 2), mesothelial cells display a cuboidal or flat morphology with scant cytoplasm and round nuclei. Nucleoli can occasionally be seen, but mitoses are absent or very few in number. An important feature is the lack of desmoplasia, inflammation, and necrosis in the stroma, a feature that denotes the absence of true infiltration.

Immunohistochemistry (Figure 2) showed that calretinin and WT-1 were both positive, demonstrating the mesothelial nature of the lesion. BAP-1 was strongly positive, as was CK7. The Ki-67 index was very low, with only a few positive nuclei. p53 was negative.

A diagnosis of florid benign mesothelial hyperplasia was made. The patient was free of disease at the last contact after 8 months of follow-up.

### 2.2. Case 2

A 62-year-old male consulted due to left scrotal enlargement. No remarkable antecedents were reported. A physical exam revealed a small left-sided hydrocele with a solid nodule. On surgery, an exophytic lesion attached to the tunica vaginalis with a narrow stalk was seen.

**Figure 3:**
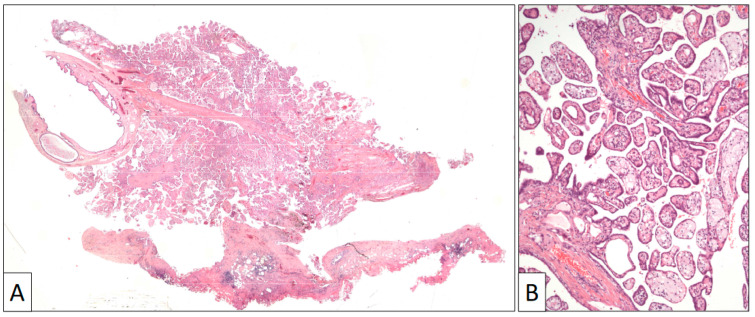
(**A**) Panoramic view of a papillary benign mesothelioma showing an exophytic architecture and absence of infiltrative growth into the tunica vaginalis. (**B**) Papillary arrangement of the lesion (original magnification, ×100).

The surgical specimen consisted of an irregular fragment of tunica vaginalis that contained a villous exophytic lesion 1 cm in diameter (Figure 3). A preliminary diagnosis of a papillary mesothelial proliferation, undetermined for malignancy, was performed in the intra-operative study. A closer microscopic view to this lesion showed an intricated proliferation of papillary structures frequently filled with foamy cells and covered by a single row of flat to cuboidal cells with no atypia or mitoses. The tunica vaginalis to which this papillary lesion was attached consisted of a fibrous capsule with denudated surface and mild lymphocytic infiltration.

**Figure 4:**
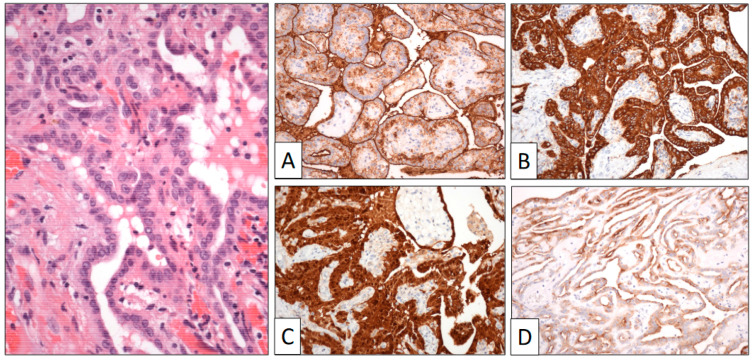
Papillary structures are covered by a single row of cuboidal cells. Immunohistochemical study shows positive reaction to HMBE-1 (**A**), calretinin (**B**), AE1-AE3 cyto-keratin (**C**), and EMA (**D**) (original magnification, ×250).

Immunohistochemistry (Figure 4) showed that calretinin, HMBE-1, and WT-1 were positive, demonstrating the mesothelial origin of the lesion. BAP-1 was strongly positive, as were cytokeratin and EMA. The proliferation index (Ki-67) was very low.

A diagnosis of well-differentiated papillary mesothelioma was made. The patient was alive and free of disease 2 years after the surgical resection.

## 3. Discussion

Mesothelial proliferations of the tunica vaginalis testis are infrequent lesions in the routine work-up of general pathologists. Their scarcity, together with their varied spectrum of morphologies, may make their correct identification a diagnostic challenge [13,14]. In this particular context, the main concerns are either missing malignancy or making an overdiagnosis. The pseudo-infiltrative growth in the first case in this report, and the complex architecture of the second exemplify this tight-rope walk. Nonetheless, true malignant mesotheliomas of the tunica vaginalis testis are rare [3]. Indeed, Butnor et al. [5] found only 18 malignant mesotheliomas in a retrospective review of more than 4000 mesothelial lesions.

The clinical context may be of help, since benign mesothelial proliferations are frequently associated with hydrocele, sometimes recurrent, as the only clinical manifestation [13]. To date, these lesions have not been associated with asbestos exposure, so the differential diagnosis with malignant mesothelioma matters and may have medico-legal implications. Blood flow with Doppler color ultrasound may be useful to preoperatively identify such inflammatory/reactive lesions. In particular, such diagnoses should be considered if low-echo light masses within the hydrocele, multiple nodular masses in the scrotum wall, or local thickening of para-epididymal sheath are detected. The physical exploration and serum tumor markers are unremarkable. On surgery, the tunica vaginalis may appear hemorrhagic and/or edematous secondary to the hydrocele, but hard fibrous thickening and attachment to neighboring structures, which would be indicative of malignant infiltration, are lacking. At most, an exophytic polyp with a villous surface such as that of the second case in this report may be observed.

Under the microscope, conventional hematoxylin–eosin-stained sections provide the first diagnostic keys. Churg et al. [1] provide a detailed histological study that distinguishes between benign and malignant mesothelial proliferations in the pleura and peritoneum. Despite all this, a grey morphological zone does exist. More specifically, mesothelial lesions in the tunica vaginalis testis may show rare examples with uncertain malignant potential [15] and benign cases mimicking malignant mesotheliomas [4].

Flat lesions like the first case in this analysis do not significantly thicken the tunica because a hard stromal reaction is mostly lacking. In fact, there is no desmoplasia in the wall. Acute and chronic inflammation may be present in varied amounts depending on the evolution of the accompanying hydrocele [13]. In this more or less inflammatory context, a mesothelial proliferation with a non-branched tubulo-papillary architecture is observed. The inner surface of the tunica vaginalis may be partially or totally denudated and substituted by a thin fibrin layer. Flattened tubules of mesothelial cells appear just beneath the surface arranged parallel to the surface. 

Exophytic lesions like the second case in this study show an arborescent architecture. Papillae in these cases are covered by a single mesothelial cell layer. Foamy histiocytes fill in the stalks. An infiltrative pattern into the wall is usually lacking. These lesions appear in the literature as well-differentiated mesotheliomas [16,17,18,19,20,21]. 

A detailed view of the mesothelial cells in flat and exophytic lesions may display flattened to cuboidal elements with reactive changes such as enlarged nuclei and occasional nucleoli, but true atypia is not seen. Mitoses are very scarce or lacking, and there is no tumor necrosis.

Immunohistochemistry is useful in two different settings: first, in confirming the mesothelial nature, and second, in confirming the benign/malignant condition of the lesions. A long list of antibodies including different keratins, BAP-1, calretinin, HBME-1, GLUT-1, CDKN2A, EMA, IMP3, vimentin, WT-1, thrombomodulin, CEA, bcl-2, P-glycoprotein, PDGF-R β-chain, desmin, p53, Ki67, and still others has been used. To make matters simpler in this clinical setting, the mesothelial nature of a lesion can be corroborated by calretinin [22], HMBE-1 [23], and WT-1 [23] positivity and its benign nature by BAP-1 positivity [10,11] and/or GLUT-1 negativity [7,8,9]. 

Last but not least, molecular analyses may provide additional information in making the differential diagnosis. In this sense, 9p21 deletion, among other deletions detected by FISH in malignant mesotheliomas [12], is not detected in benign mesothelial proliferations [4].

## 4. Conclusions

Benign mesothelial proliferations of the tunica vaginalis testis include a broad spectrum of morphologies which may pose diagnostic difficulties for general pathologists. Flat and exophytic lesions may appear, usually in the clinical context of hydrocele. This report reviews their distinctive morphological and immunohistochemical features. 

## Data Availability

All data are contained within the article.
